# Lifestyle Experiences and Coping During the COVID-19 Pandemic: A Cross-sectional Study from Saudi Arabia: Coping During Pandemic

**DOI:** 10.31661/gmj.v14i.3893

**Published:** 2025-09-10

**Authors:** Mahmoud Abdulrahman Mahmoud

**Affiliations:** ^1^ Department of Family and Community Medicine, College of Medicine, Imam Muhammad Ibn Saud University, Saudi Arabia

**Keywords:** Coping Strategies, Cross-sectional Study, Lifestyle Changes, Mental Well-being, Pandemic Response, Social Media Recruitment

## Abstract

**Background:**

The COVID-19 pandemic has prompted significant lifestyle changes worldwide. Despite extensive research on negative impacts, there’s a gap in understanding positive transitions; thus, this study explores adaptive behaviors and coping strategies during the pandemic in Saudi Arabia.

**Materials and Methods:**

A cross-sectional study recruited Saudi residents aged 18-70 via social media. Data collected through an electronic survey tool was analyzed using SPSS, focusing on demographic, health-related, and research-specific responses. Coping behaviors were interpreted using Lazarus and Folkman’s model and Carver’s Brief COPE framework.

**Results:**

Among 3,508 participants, 38.0% engaged in language learning and 55.6% read books or listened to audiobooks at a frequency of 1–3 times per month. Home cooking was reported by 79.2%, and 34.8% engaged in daily remote social interactions. Gender and nationality significantly influenced coping behaviors: reading books or audiobooks was more common among females (P0.001) and non-Saudis (P0.001), while exercising 2–4 days per week was also more prevalent among females (P=0.013). Programming interest was significantly higher among males and Saudi participants (P=0.035).

**Conclusion:**

Study Findings show that participants adopted a variety of adaptive coping strategies, such as book reading, exercise, social interaction, and engagement with online content. Framing these behaviors through established psychological coping models enhances understanding of their role in mental well-being. These findings can inform public health strategies to promote positive coping mechanisms and support mental well-being in Saudi Arabia and similar contexts.

## Introduction

The COVID-19 pandemic has been a catalyst for significant lifestyle changes worldwide. As societies went into lockdown, individuals faced new challenges and adapted to their daily routines. Research has documented these shifts, emphasizing the importance of understanding their broader implications. While many studies focus on mental health deterioration and stress-related behaviors, fewer have examined the emergence of positive coping mechanisms during this global crisis. For example, studies have shown that adults in Cyprus experienced disruptions in lifestyle habits, including diet, physical activity, increased digital interaction, and stress levels during the lockdown [1][2][3]. Similar findings were noted in Saudi Arabia [4][5], yet the focus remains largely on negative outcomes [4][5][6].

This study looks at the positive lifestyle changes people made during the lockdown and explains them using Lazarus and Folkman’s Transactional Model of Stress and Coping (1984), which separates ways of dealing with stress into problem-focused and emotion-focused coping, and Carver’s Brief COPE framework, which shows helpful versus unhelpful strategies. Understanding how individuals positively adapted during the lockdown resilience and mental well-being support strategies [7][8].

In the Gulf region, particularly in Saudi Arabia, the pandemic's influence on lifestyle has been profound. A cross-sectional study evaluated the dietary and physical activity behaviors of Saudi Arabia's adult population during the COVID-19 quarantine, revealing significant changes [4]. Another study focused on young Saudi women, examining lifestyle changes associated with COVID-19 quarantine through a prospective lens [6].

Despite the wealth of research, there is a notable gap in the literature regarding the positive aspects of lifestyle changes during the pandemic [9]. Most studies have concentrated on the negative impacts, or the challenges faced by individuals [10][11]. Our study seeks to address this gap by exploring the positive transitions in daily life practices during the lockdown period in Saudi Arabia. We aim to shed light on the adaptive behaviors and coping strategies that emerged, contributing a new dimension to the existing body of knowledge.

This research will not only fill a critical gap in literature but also provide valuable insights into the resilience and adaptability of individuals during unprecedented times. By focusing on the positive outcomes, we hope to offer a different perspective on the pandemic's impact on daily life and well-being.

## Materials and Methods

A cross-sectional study was conducted, with participants recruited aged 18 years and above through promoted social media posts. Informed consent was obtained from all participants electronically. The survey ensured strict anonymity, preventing re-identification and safeguarding participants' privacy.

The study included male and female residents. Participants who did not meet the age criteria (i.e., under 18) were excluded. Data was collected using an electronic survey tool, ensuring standardization and ease of response for participants.

The survey was designed to gather demographic information, health-related data, and responses to specific research questions. It underwent content validation by a panel expert in public health and behavioral Science. We calculated both the Content Validity Ratio (CVR≥0.78) and Item-Level Content Validity Index (I-CVI=0.87 average). A convenience sampling method was employed, leveraging the accessibility and reach of social media platforms to recruit participants. The survey remained open for 3 weeks, yielding 3,508 completed responses This method allowed for the inclusion of a diverse range of individuals from various demographic backgrounds within the Country population.

A comprehensive statistical analysis was conducted on the dataset, encompassing both descriptive and inferential methodologies. Firstly, a descriptive analysis is conducted to summarize the demographic characteristics of the participants, which include age, gender, and other features.

This process provides an overview of the study population. Subsequently, inferential analyses such as Fisher’s Exact Test are used to examine the association between categorical variables. Statistical significance is established at a p-value of 0.05 or lower and a 95% confidence interval. All statistical analyses are executed using IBM's SPSS software, version 29.0.0.

### Ethical Approval

Ethical approval for this study was obtained from the Institutional Review Board (IRB) research ethical committee of Research Deanship, Imam Muhammad Ibn Saud Islamic University under project number 565/2023 in accordance with the Declaration of Helsinki.

## Results

**Table:** Table1. Participants Demographic Data

Variable		Frequency (n=3508)	Percent (%)
Age	Mean SD Range	34.71 years 9.32 years 18-78 years	
Gender	Male	1,493	42.56
	Female	2,015	57.44
Nationality	Saudi	2,928	83.47
	Non-Saudi	580	16.53
	Less than high school	4	0.11
Education	High school	150	4.28
	University	2,687	76.60
	Higher education	667	19.01
	Student	185	5.27
	Public sector employee	2,030	57.87
Job	Working in private sector	728	20.75
	Retired	35	1.00
	Not working	530	15.11
	Yes (in person)	1,861	53.05
	Yes (online)	549	15.65
Working during the lockdown	Formal vacation	171	4.87
	I was not working	927	26.43
You or a family member infected with COVID-19 affected your life	Yes	1,739	49.57
	No	1,769	50.43

**Table:** Table2. Participants Source of Information and Time Duration about COVID-19

Source of information		Frequency (n=3508)	Percent (%)
Official Sites	Yes	2,884	82.21
	No	624	17.79
Social media	Yes	2,356	67.16
	No	1,152	32.84
Family or Friends	Yes	1,047	29.85
	No	2,461	70.15
Information as work in health sector	Yes	43	1.23
	No	3,465	98.77
Others	Yes	5	0.14
	No	3,503	99.86
	Less than 1 hour	1,561	44.50
Time spent in following COVID-19 news daily	1-2 hours	973	27.74
	2-4 hours	480	13.68
	More than 4 hours	494	14.08

**Table:** Table4. Gender and Nationality Differences in Coping Behaviors

Behavior	Higher in…	p-value
Reading books/audiobooks	Females, Non-Saudis	< 0.001
Exercising 2-4 days/week	Females	<0.013
Cooking	Females	< 0.001
Listening to podcasts	Females, Non-Saudis	< 0.001
Reading religious books	Males, Non-Saudis	< 0.001
Reading social science books	Females, Saudis	< 0.001
Programming interest	Males, Saudis	< 0.035
Health/medical book reading	Non-Saudis	< 0.001
Remote social interaction	Females	<0.01
Sleeping 6-8 hours	Males, Non-Saudis	< 0.01

^*^P<0.05 ^*^Statistical significance determined by Fisher’s Exact Test.

Our study included 3508 participants (Table-1). Gender distribution showed 42.56% (n=1493) were males and 57.44% (n=2015) were female. The mean age was 34.71 years, with a standard deviation (SD) of 9.32 years and a range of 18-78 years. Regarding nationality, 83.47% (n=2928) were Saudi, while 16.53% (n=580) were non-Saudi. Education levels varied, with 0.11% (n=4) having less than high school, 4.28% (n=150) high school, 76.60% (n=2687) university, and 19.01% (n=667) higher education. In terms of employment, 5.27% (n=185) were students, 57.87% (n=2030) worked in the public sector, 20.75% (n=728) in the private sector, and 15.11% (n=530) were not working. During the lockdown, 53.05% (n=1861) worked in person, 15.65% (n=549) online, 4.87% (n=171) were on formal vacation, and 26.43% (n=927) were not working. Additionally, 49.57% (n=1739) reported being affected by COVID-19, while 50.43% (n=1769) were not.

Participants learn about COVID-19 from various sources with different volumes of time, as shown in Table-2, which outlines participants' sources of information and time spent following COVID-19 news. The majority (82.21%, n=2884) relied on official sites, while 17.79% (n=624) did not. Social media served as a source for 67.16% (n=2356) but not for 32.84% (n=1152). Family or friends provided information for 29.85% (n=1047). A minority (1.23%, n=43) obtained information through work in the health sector. Only 0.14% (n=5) relied on other sources. Regarding time spent daily on COVID-19 news, 44.50% (n=1561) spent less than 1 hour, 27.74% (n=973) spent 1-2 hours, 13.68% (n=480) spent 2-4 hours, and 14.08% (n=494) spent more than 4 hours. During the COVID-19 lockdown, participation in various lifestyle and coping activities was recorded across multiple domains. In the learning category, 55.6% (1,950) reported reading books or audiobooks (1-3/month), 38.0% (1,332) engaged in language learning, and 15.1% (531) learned sign language. Regarding exercise habits, 40.2% (n=1,409) exercised 2-4 days per week, whereas for session time to exercise, 34.95% (n=1,226) had exercise sessions lasting 15-30 minutes. In creative engagement included reading of any amount by 76.5% (2,685), gardening or plant care by 43.6% (1,529), and podcast listening (≥1/month) by 34.0% (1,192). In terms of daily routines, 79.2% (2,777) cooked at home, 34.8% (1,219) had daily remote social interactions, For social interaction, 34.75% (n=1,219) engaged in remote interactions daily and 25.7% (903) wrote in a diary at least once per week. For entertainment, 54.6% (1,915) watched documentaries (≥1/month), while only 9.4% (331) visited virtual museums. Under other activities, 50.8% (1,783) participated in volunteer work for at least one hour per week.

**Table:** Table3. Lifestyle and Coping Activities During Lockdown

Activity Domain	Behavior	n	%
Learning	Language learning	1,332	38.0%
	Book/audiobook reading (1-3/month)	1,950	55.6%
	Sign language learning	531	15.1%
Physical Activity	Exercised 2-4 days/week	1,409	40.2%
	Exercise sessions 15-30 mins	1,226	34.9%
Creative Engagement	Reading during lockdown (any amount)	2,685	76.5%
	Gardening/plant care	1,529	43.6%
	Podcast listening (≥1/month)	1,192	34.0%
Daily Routines	Learning ooking at home	2,777	79.2%
	Remote social interaction (daily)	1,219	34.8%
	Diary writing (≥1/week)	903	25.7%
Entertainment	Virtual museum visits (any)	331	9.4%
	Documentary watching (≥1/month)	1,915	54.6%
Other	Volunteer work (≥1 hr/week)	1,783	50.8%
Sleep Patterns	During daylight hours In the evening	951 2,557	27.11 72.89

Note: Participants could select multiple responses.

The most common sleep pattern was in the evening, with 2,557 (72.89%) individuals adopting this routine. Moreover, during the COVID-19 lockdown, certain predominant trends emerged among participants across various activities. Table-4 shows participants' activities in relation to gender and nationality during the COVID-19 pandemic. Reading books or audiobooks was significantly more common among females (P<0.001) and non-Saudi participants (P<0.001). Similarly, podcast listening was higher among females and non-Saudis (P<0.001).

Cooking was more frequently reported by females (P<0.001), while exercising 2-4 days per week was also significantly higher among females (P=0.013). In contrast, reading religious books was more common among males and non-Saudis (P<0.001), while reading social science books was reported more by females and Saudi participants (P<0.001). Interest in programming was significantly higher among males and Saudi participants (P=0.035). Reading health and medical books was more prevalent among non-Saudis (P<0.001). A higher proportion of females engaged in daily remote social interaction compared to males (P<0.01). Regarding sleep, more males and non-Saudi participants reported sleeping 6-8 hours per day (P<0.01). These results demonstrate statistically significant differences in coping strategies based on gender and nationality during the lockdown.

## Discussion

The purpose of this research was to look at the beneficial changes people made to their daily routines during the COVID-19 lockdown and interpret them through established coping frameworks. We wanted to see how people adjusted to the new situation caused by the pandemic. According to Lazarus and Folkman’s model, behaviors such as language learning, cooking, and organizing routines represent problem-focused coping—deliberate actions to manage stressors. Other behaviors, like reading, exercising, socializing online, or gardening, align more with emotion-focused coping, aimed at emotion regulation and psychological comfort.

The results show that many people took up new hobbies, got more exercise, and read more books to cope with the situation. A significant majority of participants did not engage in learning a new language or sign language during the COVID-19 lockdown. This could suggest a prioritization of other activities or a lack of resources or motivation for language acquisition in a crisis. In contrast, a study by Henderson et al. (2023) found an increase in language learning, indicating a possible cultural or situational difference [12].

Over half of the participants read between 1-3 books per month, showing a moderate engagement with literature. This is consistent with findings by Alomari et al. (2023), who reported similar reading habits during lockdown periods [13]. The preference for social science books could reflect a desire to understand the societal impacts of the pandemic [14]. Using Carver’s Brief COPE Inventory, the majority of these are adaptive coping strategies that promote resilience. For example, participants using reading or gardening as mood regulation tools reflect positively on emotional adjustment. While few participants engaged in maladaptive behaviors (e.g., excessive screen time or avoidance), the high rates of proactive activities suggest a population-wide tendency toward constructive coping. Gender and cultural differences may shape coping preferences, underlining the need for culturally sensitive interventions. For example, Saudi participants preferred social sciences, while non-Saudis engaged more in podcasts and data science content. The findings align with global research [10][15] and offer suggestions for how communities manage uncertainty.

The data indicates that a moderate percentage of participants exercised 2-4 days per week, suggesting that physical health was a concern for many during the lockdown [16]. This finding aligns with Abdelbasset et al. (2021), who noted the importance of maintaining physical activity for mental and physical well-being during confinement [17].

A small but significant portion of participants listened to podcasts, possibly as a form of entertainment or education during the lockdown. This is slightly lower than the trends observed by Lindgren et al. (2024), where podcast listening was a popular activity, possibly due to regional differences in content availability and preferences [18].

The majority of participants did not visit virtual museums, which could be due to a lack of interest or awareness of such resources. This contrasts with the study by Pourmoradian et al. (2021), which saw a surge in virtual museum visits, suggesting that promotional efforts may influence participation rates [19].

A high percentage of participants engaged in learning cooking during the lockdown, which may reflect the necessity of home cooking due to restaurant closures, as well as the opportunity for skill development. This is in line with the findings of Piochi et al. (2022), who observed an increase in home cooking as a leisure activity [20].

A significant portion of participants engaged in daily remote interactions, highlighting the importance of social connectivity during isolation. This mirrors the research by James et al. (2023), which emphasized the role of technology in maintaining social ties during the pandemic [21].

Gardening was a popular activity among participants, possibly as a way to stay active and connected with nature during lockdown. This finding is supported by the work of Theodorou et al. (2021), who found that gardening provided emotional relief and a sense of accomplishment during uncertain times [22].

This study holds important clinical implications for healthcare professionals and policymakers in Saudi Arabia and beyond. Understanding the diverse range of activities individuals engaged in during the COVID-19 lockdown can inform tailored interventions aimed at promoting mental health and well-being [23]. For instance, promoting activities such as book reading, exercise, and social interaction could be integrated into mental health support programs to enhance coping mechanisms and resilience during periods of crisis [23][24]. Furthermore, the significant differences observed in activity preferences based on gender and nationality underscore the need for culturally sensitive and gender-specific interventions to address the unique needs and preferences of diverse populations. [19].

Future research could build upon these findings by exploring the longitudinal effects of these activities on mental health outcomes, investigating the role of specific activities in mitigating stress and anxiety, and identifying barriers to engagement in beneficial activities among different demographic groups. Qualitative studies could provide deeper insights into individuals' subjective experiences and motivation for engaging in specific activities during times of adversity. Such research efforts would contribute to the development of more targeted and effective interventions to support individuals' mental health and well-being during future crises.

## Conclusion

This study reveals a wide array of lifestyle adaptations during the COVID-19 pandemic in Saudi Arabia. Interpreting these changes through psychological coping frameworks highlights their role in supporting mental well-being. Significant differences in events based on gender and nationality highlight the importance of tailoring interventions to meet the unique needs and preferences of different demographic groups. The findings emphasize the importance of promoting adaptive, culturally relevant behaviors—such as reading, physical activity, and virtual engagement—as part of public health responses to crises. By understanding the factors influencing individuals' engagement in various activities, healthcare professionals and policymakers can better support communities in Saudi Arabia and beyond in maintaining positive mental health outcomes during times of uncertainty and adversity.

## Conflicts of Interest

The author declares no conflicts of interest.
